# Predictive Performance of Machine Learning-Based Methods for the Prediction of Preeclampsia—A Prospective Study

**DOI:** 10.3390/jcm12020418

**Published:** 2023-01-04

**Authors:** Alina-Sinziana Melinte-Popescu, Ingrid-Andrada Vasilache, Demetra Socolov, Marian Melinte-Popescu

**Affiliations:** 1 Department of Mother and Newborn Care, Faculty of Medicine and Biological Sciences, ‘Ștefan cel Mare’ University, 720229 Suceava, Romania; 2 Department of Obstetrics and Gynecology, ‘Grigore T. Popa’ University of Medicine and Pharmacy, 700115 Iasi, Romania; 3 Department of Internal Medicine, Faculty of Medicine and Biological Sciences, ‘Ștefan cel Mare’ University, 720229 Suceava, Romania

**Keywords:** preeclampsia, prediction, machine leaning, pregnancy, first trimester

## Abstract

(1) Background: Preeclampsia (PE) prediction in the first trimester of pregnancy is a challenge for clinicians. The aim of this study was to evaluate and compare the predictive performances of machine learning-based models for the prediction of preeclampsia and its subtypes. (2) Methods: This prospective case-control study evaluated pregnancies that occurred in women who attended a tertiary maternity hospital in Romania between November 2019 and September 2022. The patients’ clinical and paraclinical characteristics were evaluated in the first trimester and were included in four machine learning-based models: decision tree (DT), naïve Bayes (NB), support vector machine (SVM), and random forest (RF), and their predictive performance was assessed. (3) Results: Early-onset PE was best predicted by DT (accuracy: 94.1%) and SVM (accuracy: 91.2%) models, while NB (accuracy: 98.6%) and RF (accuracy: 92.8%) models had the highest performance when used to predict all types of PE. The predictive performance of these models was modest for moderate and severe types of PE, with accuracies ranging from 70.6% and 82.4%. (4) Conclusions: The machine learning-based models could be useful tools for EO-PE prediction and could differentiate patients who will develop PE as early as the first trimester of pregnancy.

## 1. Introduction

Preeclampsia (PE) is a complex condition associated with pregnancy that could lead to important feto-maternal morbidity and mortality. The subclassification of PE includes: (a) early-onset PE (EO-PE), with delivery at <34 + 0 weeks of gestation; (b) late-onset PE (LO-PE), with delivery at ≥34 + 0 weeks of gestation; (c) preterm PE, with delivery at <37 + 0 weeks of gestation; (d) and term PE, with delivery at ≥37 + 0 weeks of gestation [1]. The incidence of preeclampsia varies among different regions of the world, and a recent meta-analysis estimated a global incidence of 4.6 % (95 % confidence interval (CI): 2.7–8.2) for this disorder [2]. PE is more widespread in developing nations, where the prevalence ranges between 1.8 and 16.7% [3,4]. 

Among the most frequently cited adverse pregnancy outcomes for patients affected by preeclampsia are stillbirth, preterm birth, intrauterine growth restriction, low Apgar scores, and higher admission rates in the neonatal intensive care units of the newborns [5,6]. Moreover, these adverse pregnancy outcomes appear to be more severe for the early-onset form of preeclampsia [1,7,8]. Therefore, it is important to identify women at risk of developing PE as early as the first trimester, considering that the administration of aspirin is necessary before 16 weeks of gestation in order to prevent this disorder and its complications [9,10]. 

Numerous screening strategies have been developed over time in order to obtain the best results in terms of the predictive performance of the various approaches. Thus, researchers have used maternal characteristics, the mean arterial pressure (MAP), serum biomarkers, and the mean uterine artery pulsatility index (UTPI) measured in the first trimester of pregnancy as key parameters included in the screening process [11]. The risk stratification used in these strategies is based on individual risk factors, logistic regression (LR), or the competing risk approach [12,13,14,15,16]. 

The risk factors for PE derived from the maternal characteristics include: advanced maternal age, Afro-Caribbean or South Asian ethnicity, nulliparity, a previous history of PE, short or long inter-pregnancy intervals, the use of assisted reproductive technologies (ART), a family history of PE, obesity, hyperglycemia in pregnancy, pre-existing chronic hypertension, renal disease, and autoimmune diseases such as systemic lupus erythematosus (SLE), and anti-phospholipid syndrome (APS) [17,18,19]. 

On the other hand, the panel of biomarkers evaluated in different screening algorithms varies considerably [20,21]. The most used biomarkers in the first trimester of pregnancy, with good predictive performance, are placental growth factor (PLGF) and pregnancy-associated plasma protein-A (PAPP-A). Three prospective non-intervention screening studies of PE in the first trimester of pregnancy, which included a combination of maternal risk factors (MAP, PLGF, and UtA-PI), using a risk cut-off of 1 in 100 for preterm PE, demonstrated that the detection rates for early-onset, preterm, and term PE were 88%, 69%, and 40%, respectively [22]. 

Placental protein-13 (PP-13) is another serum biomarker that can be incorporated in the combined first trimester screening of PE. A recent meta-analysis indicated a higher accuracy of PP-13 for the screening of late-onset preeclampsia when compared with early-onset preeclampsia [23]. Moreover, biomarkers derived from proteomic, metabolomic, and genomic studies have the potential to reveal a greater specificity for the disease, although the costs of such technologies are high [24,25,26]. 

Artificial intelligence and machine learning techniques have the potential to outperform the conventional screening strategies of preeclampsia, and to evaluate big datasets in order to provide a comprehensive picture over the heterogenous phenotypic manifestation of the disorder. Machine learning is a new field that evaluates how computers learn from data [27,28]. Computer learning can be conveniently divided into two categories: supervised learning and unsupervised learning [29]. Supervised learning starts with the goal of predicting a known output or target, while in unsupervised learning, the algorithm attempts to find naturally occurring patterns or groupings within the data [27]. The machine learning-based methods for the prediction of PE fall under the supervised category and include random forest (RF), decision trees (DT), gradient boosting (GB), naïve Bayes (NB), and support vector machine (SVM) [30,31]. Various medical data are selected as features for training and testing algorithms, and the results are interpreted in forms of predictive performance [32]. 

A recent systematic review and meta-analysis evaluated machine learning models and compared their predictive performances with logistic regression models for the predictions of pregnancy events [33]. The authors found a superior performance of machine learning models for the prediction of preeclampsia when using random forest or decision trees. Moreover, a cross-sectional study that evaluated six data mining methods for the prediction of PE in a cohort of 1452 pregnant women found that the SVM method had the highest accuracy (0.791), followed by DT (0.788) and RF (0.758) [34]. 

The aim of this study was to evaluate and compare the predictive performances of machine learning-based models for the prediction of preeclampsia and its subtypes. 

## 2. Materials and Methods

We conducted a prospective case-control study of pregnancies that occurred in women who attended a tertiary maternity hospital (‘Cuza-Voda’, Iasi, Romania) between November 2019 and September 2022. Ethical approval for this study was obtained from the Institutional Ethics Committee of the University of Medicine and Pharmacy ‘Grigore T. Popa’ (No. 151/13 February 2022). Informed consent was obtained from all participants included in the study. All methods were carried out in accordance with the relevant guidelines and regulations. 

We recruited participants at the time of the routine first trimester screening. The inclusion criteria taken into consideration were pregnant patients with singleton pregnancies, maternal age ≥ 18, and certain first trimester pregnancy dating. Exclusion criteria comprised patients who had multiple pregnancies, ectopic pregnancies, first and second trimester abortions, fetal intrauterine demise, fetuses with chromosomal or structural abnormalities, intrauterine infection, incomplete medical records, incorrect/lack of first trimester sonographic pregnancy dating, or who were unable to offer informed consent. 

Maternal characteristics and previous medical history were evaluated by a physician, and maternal risk factors for preeclampsia were recorded in the database. The following parameters were evaluated: demographic data, parity, obstetrical comorbidities, BMI (body mass index), smoking status during pregnancy, inter-pregnancy intervals, the use of ART, a personal or family history of PE, and comorbidities (hyperglycemia in pregnancy, pre-existing chronic hypertension, renal disease, SLE, and APS). 

Blood pressure was measured using the Fetal Medicine Foundation (FMF) guidelines [35] with a calibrated device (Omron M3 COMFORT; Omron Corp, Kyoto, Japan), and the mean arterial pressure was recorded. The first trimester ultrasound screening and UtA-PI evaluation was performed transabdominally according to FMF guidelines [36] by certified physicians in maternal–fetal medicine. 

Blood (serum and plasma) samples were collected before the first trimester scan and stored at −80 °C degrees for further studies. For the current study, PAPP-A and PLGF serum levels were measured using a BRAHMS Kryptor analyzer (Thermo Fisher Scientific, Germany), while PP-13 serum levels were determined using the quantitative sandwich ELISA (enzyme-linked immunosorbent assay) method. The serum levels of these biomarkers were converted to multiples of median (MoM) by logarithm. 

All pregnant women were evaluated by an experienced obstetrician with an early ultrasound scan, using an E8/E10 (General Electric Healthcare, Zipf, Austria) scanner with a 4.8 MHz transabdominal probe (GE Medical Systems, Milwaukee, WI, USA), between 10 + 0 and 13 + 6 weeks, in order to determine gestational age by measuring the crown–rump length (CRL), as well as UtA-PI. 

A total of 233 patients were included in the analysis of this study and divided into two equal groups: those who developed preeclampsia (116 patients, group 1), and those who did not develop preeclampsia (116 patients, group 2). PE was defined as the de novo development of hypertension (blood pressure ≥ 140/90 mm Hg, four hours apart) and any sign(s) of organ deficiency, including proteinuria (daily urinary protein loss ≥ 0.3 g), liver function deterioration (high transaminase levels), thrombocytopenia (platelet count ≤ 150.000/mL), or neurologic symptoms (visual sensations) appearing during the second half of pregnancy [37]. The following pregnancy outcomes were recorded: type of birth, presentation, gestational age at birth, newborn’s gender, birthweight, length, and Apgar scores at 1 and 5 min. 

In the first stage of the statistical analysis, each variable was evaluated with chi-squared and Fisher’s exact tests for categorical variables, which were presented as frequencies with corresponding percentages, and *t*-tests for continuous variables, which were presented as means and standard deviations (SD).

The pregnant patients affected by preeclampsia were subsequently divided into the following subgroups: subgroup 1 (EO-PE, n = 22), subgroup 2 (LO-PE, n = 94), subgroup 3 (moderate PE, n = 88), and subgroup 4 (severe PE, n = 28). EO-PE was defined considering the onset of the disease at less than 34 weeks of gestation, while LO-PE had an onset at or after 34 weeks of gestation [38]. Severe preeclampsia was considered in the presence of the following criteria: systolic blood pressure of 160 mm Hg or more, or diastolic blood pressure of 110 mm Hg or more on two occasions, at least 4 h apart; thrombocytopenia (<100 × 10^9^/L); renal insufficiency (serum creatinine > 1.1 mg/dl or doubling of serum creatinine in the absence of other renal disease); impaired liver function (elevated blood concentrations of liver transaminases to twice normal concentration); pulmonary edema; unexplained new-onset headache unresponsive to medication (without an alternative diagnosis); or visual disturbances [39].

An ANOVA analysis with the Bonferroni post hoc test was used to determine whether or not there was a statistically significant difference between the subgroups regarding their paraclinical characteristics (serum biomarkers, MAP, and UtA-PI), and boxplots were used for graphical representations of these differences. The statistical analyses were performed using STATA SE (version 14, 2015, StataCorp LLC, College Station, TX, USA).

In the second stage of the analysis, we evaluated the predictive performance of 4 machine learning- based models: decision tree, naïve Bayes, support vector machine, and random forest algorithm. 

One of the first and most well-known machine learning techniques is the DT, which represents the tests and outcomes for categorizing data elements into a tree-like structure [40]. A DT tree’s nodes typically have numerous layers, with the first node referred to as the root node [41,42]. All internal nodes reflect input variable or attribute testing. The classification algorithm branches towards the appropriate internal node based on the test result, and the process of testing and branching is repeated until it reaches the leaf node. The predicted outcomes are represented by the leaf or terminal nodes.

NB is a classification technique based on the Bayes’ theorem [43]. This theorem can predict the likelihood of an occurrence depending on prior knowledge of the event’s conditions. This classifier asserts that a given characteristic in a class is not directly related to any other feature, even though the features in that class may be interdependent [41].

An SVM is a supervised learning algorithm used for classification and regression [44,45]. This algorithm is a relatively new method that has shown promising results in recent years for disease prediction. SVM classifiers are based on linear classifiers and seek to select a line that is slightly more confident.

Random forests are ensemble classifiers that randomly learn multiple decision trees [46]. The random forest approach consists of a training stage in which many decision trees are built and a testing step in which an outcome variable is classified or predicted based on an input vector [41]. The different decision trees of an RF are trained using the different parts of the training dataset. To classify or predict a new sample, the input vector of that sample needs to be passed down with each DT of the forest. Each DT then considers a different part of that input vector and offers a prediction outcome. The forest then selects the prediction with the greatest number of ‘votes’ (for discrete outcomes) or the average of all trees in the forest (for numeric outcomes).

The data were segregated into data for testing (70%) and data for training (30%). In order to protect from overfitting, all models underwent 5-fold cross validation. Their true positive rates (TPR), false negative rates (FNR), positive predictive values (PPV), false detection rates (FDR), accuracies, values for area under the curve (AUC), precision, recall, and F1 scores were calculated, and compared for preeclampsia, EO-PE, LO-PE, moderate PE, and severe PE subgroups, respectively. The comparison was made using between-groups variance, measured by an ANOVA and a Bonferroni post hoc test. The models were constructed and analyzed using Matlab (version R2021b, The MathWorks, Inc., Natick, MA, USA). 

## 3. Results

A total of 233 pregnant patients were evaluated in our prospective study. Their clinical and paraclinical characteristics are presented in Table 1 and are segregated into the following groups: preeclampsia (116 patients, group 1), without preeclampsia (group 2, 116 patients). The preeclampsia group contained significantly more patients with a personal history of hypertension (*p* = 0.005) and preeclampsia in previous pregnancies (*p* < 0.001). Moreover, obesity was more prevalent in the first group compared to the second group (*p* < 0.001). Regarding the paraclinical characteristics measured in the first trimester of pregnancy, the MAP, UtA-PI, and PLGF were significantly higher for the PE group, while PP-13 and PAPP-A were significantly lower for this group (*p* < 0.001). 

The pregnancy outcomes for the main groups are presented in Table 2. Pregnancies affected by PE were significantly associated with complications such as preterm birth (*p*< 0.001), intrauterine growth restriction (*p* < 0.001), and oligoamnios (*p* = 0.01). Eclampsia, abruptio placentae, and HELLP syndrome (Hemolysis, Elevated Liver enzymes and Low Platelets) had a low incidence in group 1 of patients, and none of them manifested in the second group, mainly because they are specifically associated with this disorder. 

The patients in the PE group had a significantly higher cesarean delivery rate (n = 112 patients, 96.55%; *p* < 0.001), and their newborns had a significantly lower birthweight, Apgar scores at 1 and 5 min, and length (*p* < 0.001). 

**Figure 1:**
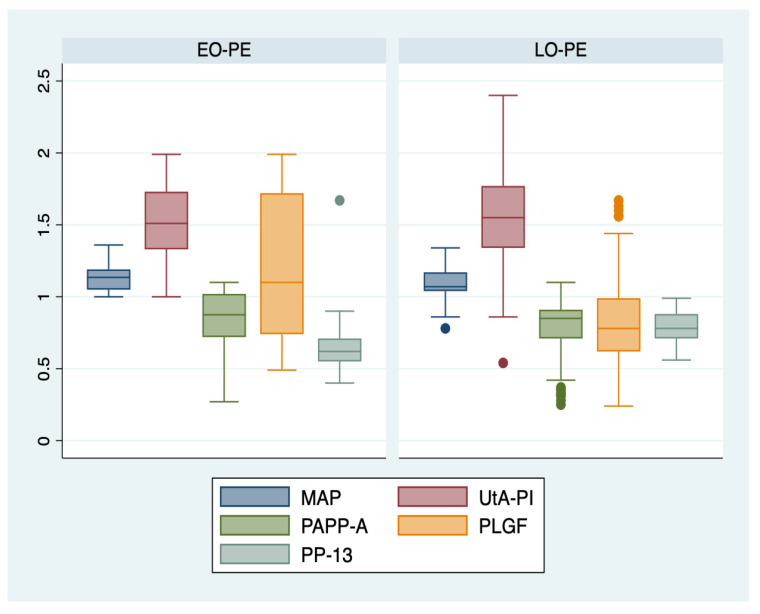
Boxplot representing the comparison of paraclinical parameters in subgroups 1 and 2. Legend: MAP—mean arterial pressure; UtA-PI—uterine artery pulsatility index; PLGF—placental growth factor; PP-13—placental protein-13; PAPP-A—pregnancy-associated plasma protein-A; EO-PE—early- onset preeclampsia; LO-PE—late-onset preeclampsia.

**Figure 2:**
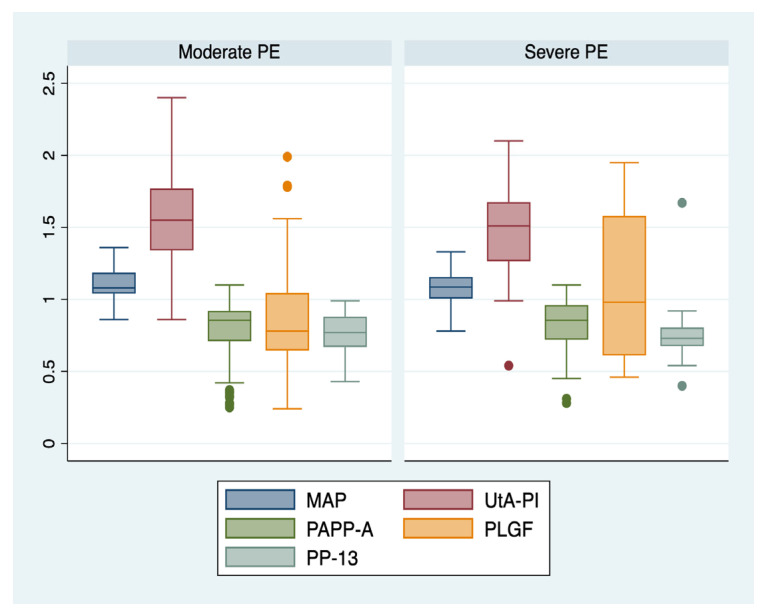
Boxplot representing the comparison of paraclinical parameters in subgroups 3 and 4. Legend: MAP—mean arterial pressure; UtA-PI—uterine artery pulsatility index; PLGF—placental growth factor; PP-13—placental protein-13; PAPP-A—pregnancy-associated plasma protein-A; PE—preeclampsia.

We further comparatively analyzed the paraclinical characteristics of the following subgroups: EO-PE (22 patients, subgroup 1), LO-PE (n = 94, subgroup 2), moderate preeclampsia (n = 88, subgroup 3), severe preeclampsia (n = 28, subgroup 4) (Table 3). The serum values of PLGF determined in the first trimester of pregnancy were significantly higher for the EO-PE and severe PE subgroups (*p* < 0.001), while the serum levels of PP-13 were significantly lower for the LO-PE subgroup (*p* = 0.003). A graphical representation of the comparison is represented in Figure 1 and Figure 2. 

In the second stage of the analysis, we incorporated the pregnant patient’s clinical and paraclinical characteristics into four machine learning-based models, and we calculated their predictive performance (Table 4). DT achieved the highest accuracy when predicting the EO-PE group (94.1%), with an AUC value of 0.95, while its highest TPR was achieved for all types of preeclampsia prediction. The NB model had the highest performance when used to predict all types of PE, with an accuracy of 98.6%, and an AUC value of 0.98. A similar situation described the predictive performance of the RF model, which achieved an accuracy of 92.8%, with an AUC value of 0.94 for all types of preeclampsia. Finally, the SVM model appeared to have the highest predictive performance when used to predict EO-PE patients, achieving an accuracy of 91.2%, and an AUC value of 0.91. DT and RF had the highest TPR for all types of preeclampsia prediction (94.1%), while SVM and NB were characterized by a high TPR (96.4%) when used to predict EO-PE. 

We analyzed the variance between preeclampsia groups, taking into consideration the predictive parameters from the machine learning-based models (Table 5). Our results showed significant variance between groups for all the parameters (*p* < 0.001). The large F value indicates that the means of the groups are greatly different from each other compared to the variation of the individual observations in each group and support the hypothesis that the differences between group means are larger than what would be expected by chance.

## 4. Discussion

This is the first prospective study in the literature that trained four machine learning-based models (DT, NB, SVM, and RF) for the prediction of preeclampsia in a cohort of pregnant patients with singleton pregnancies, using clinical and paraclinical parameters determined in the first trimester. Other particularities of this study are that we included the serum levels of PP-13, expressed as MoM in the analysis, and we calculated the predictive performance of these models for preeclampsia subtypes. 

Our results showed that EO-PE was best predicted by DT and SVM models, while NB and RF models had the highest performance when used to predict all types of PE. The predictive performance of these models was modest for moderate and severe types of PE subgroups, with accuracies ranging from 70.6% and 82.4%. 

Regarding the prediction of LO-PE, the highest accuracy was achieved by DT and RF models (88.2%), with AUC values of 0.80 and 0.84, respectively. A recent retrospective study that evaluated the predictive performance of six ML-based models for LO-PE in a cohort with singleton pregnancies, and used clinical and paraclinical parameters determined as early as the second trimester of pregnancy, indicated similar performances for DT (C- statistic: 0.857) and RF models (C- statistic: 0.894), and higher performances for the stochastic gradient-boosting model (C- statistic: 0.924) [30]. However, the above-mentioned study included repeated common laboratory measurements in the analysis, while we included the biomarkers recognized in the literature as predictors of PE [11,47,48]. 

Additionally, our results showed that the serum values of PLGF determined in the first trimester of pregnancy, expressed as MoM, were significantly higher for the EO-PE, and severe PE subgroups (*p* < 0.001), while the serum levels of PP-13 were significantly lower for the LO-PE subgroup (*p* = 0.003). These findings are in line with data published in the literature that confirmed the superior predictive performance of PLGF for early onset and severe types of PE [49,50,51], and of PP-13 for LO-PE [23]. 

Many of the existing models for predicting preeclampsia are risk score models that are based on epidemiological data and/or clinical factors [14,18,21,52,53]. In a prospective study by Di Lorenzo et al. evaluating the detection of preeclampsia by integrating maternal history, serum biomarkers, and uterine artery Doppler in the first trimester, the authors reported a sensitivity of 60% (TPR) for a 20% FPR for all types of PE when using a combination of UtA-PI, PlGF, and a maternal history of chronic hypertension [47]. Our combined models achieved higher sensitivities for all types of preeclampsia (TPR ranges: 70.6–96.3%).

A recent unicentric study on 498 patients, which evaluated the results from the first trimester screening of PE in a 5 years’ time frame, revealed that an algorithm based on risk factors from the maternal history, the serum level of PlGF and PAPP-A, the calculated value of MAP, and the measured values of the uterine arteries PI achieved a PPV for early PE of 21.3% [54]. We obtained higher PPV (range: 71.4–80%) for EO-PE prediction using the proposed machine learning-based methods, and these results could be due to the inclusion of PP-13 as a biomarker that has high sensitivity for EO-PE [55].

Our study has several limitations, including a small cohort of patients and number of predictors, but at the same time, the trained models have the advantage of easier implementation by physicians. All chosen machine learning-based models have the ability to handle small sample data. We hypothesize that the model’s accuracy could be improved by adding repeated measurements during pregnancy of the paraclinical parameters, as well as the sFlt-1 (soluble fms-like tyrosine kinase 1)/PLGF ratio, which has been proven as a useful biomarker for PE prediction in the second trimester of pregnancy [56,57,58]. 

Further studies, on larger cohorts of patients, could evaluate the predictive performance of these ML-based models in different settings and populations. The results could aid clinicians in the risk stratification process of pregnant patients as early as the first trimester and could help calculate the risk–benefit ratio in order to support the decision of PE prophylaxis with aspirin, 150 mg/night, from 11–14 until 36 weeks of gestation [1]. 

## 5. Conclusions

The machine learning-based models could be useful tools for EO-PE prediction and could differentiate patients who will develop PE as early as the first trimester of pregnancy.

These finding are important for clinicians because very often the early-onset form of preeclampsia needs an individualized management of delivery, which in most cases is recommended prematurely, before 37 weeks of gestation, adding supplementary distress to the newborn.

Moreover, the proposed methods showed good results for differentiating patients who will develop preeclampsia later in pregnancy from patients who will not develop this disease. This information could support clinicians’ decision of aspirin prophylaxis early in pregnancy.

## Figures and Tables

**Table 1:** Clinical and paraclinical characteristics of the patients included in the main groups.

Patient’s Characteristics	Group 1 (PE, n = 116)	Group 2 (Without PE, n = 116)	*p* Value
Age, years (mean ± SD)	29.22 ± 6.88	28.62 ± 6.39	0.49
BMI (kg/m^2)^	26.4 ± 1.07 **	21.87 ± 2.47	<0.001
Medium (n/%)	Urban = 54 (46.55%) Rural = 62 (53.45%)	Urban = 53 (45.69%) Rural = 63 (54.31%)	0.10
Parity (n/%)	Nulliparity = 75 (64.66%) Multiparity = 41 (35.34%)	Nulliparity = 65 (56.03%) Multiparity = 51 (43.97%)	0.18
Personal history of PE (n/%)	Yes = 10 (8.62%) **	Yes = 0 (0%)	<0.001
Personal history of hypertension (n/%)	Yes = 10 (8.62%) **	Yes = 1 (0.86%)	0.005
Personal history of renal disease (n/%)	Yes = 2 (1.72%)	Yes = 0 (0%)	0.15
Personal history of diabetes (n/%)	Yes = 2 (1.72%)	Yes = 0 (0%)	0.15
Personal history of SLE/APS (n/%)	Yes = 9 (7.76%)	Yes = 3 (2.59%)	0.07
Obesity (n/%)	Yes = 31 (26.72%) **	Yes = 5 (4.31%)	<0.001
Interpregnancy interval, years (mean ± SD)	1.02 ± 1.93	1.13 ± 1.85	0.65
MAP, MoM (mean ± SD)	1.10 ± 0.10 **	0.85 ± 0.15	<0.001
UtA-PI, MoM (mean ± SD)	1.54 ± 0.29 **	0.82 ± 0.21	<0.001
PAPP-A, MoM (mean ± SD)	0.82 ± 0.52 **	1.00 ± 0.16	<0.001
PLGF, MoM (mean ± SD)	2.17 ± 0.96 **	1.35 ± 0.34	<0.001
PP-13, MoM (mean ± SD)	0.75 ± 0.12 **	1.09 ± 0.13	<0.001

Table 1 legend: PE—preeclampsia; SD—standard deviation; APS—antiphospholipid syndrome; SLE—systemic lupus erythematosus; MoM—multiples of median; MAP—mean arterial pressure; UtA-PI—uterine artery pulsatility index; PLGF—placental growth factor; PP-13—placental protein-13; PAPP-A—pregnancy-associated plasma protein-A. Tests used: chi-squared for categorical variables, and *t*-tests for continuous variables; ** The data from which statistical significance originates.

**Table 2:** Pregnancy outcome of the patients included in the main groups.

Pregnancy Outcome	Group 1 (PE, n = 116)	Group 2 (Without PE, n = 116)	*p* Value
Placenta praevia (n/%)	Yes = 5 (4.31%)	Yes = 7 (6.03%)	0.55
Preterm birth (n/%)	Yes = 51 (43.97%) **	Yes = 5 (4.31%)	<0.001
Intrauterine growth restriction (n/%)	Yes = 48 (41.38%) **	Yes = 5 (4.31%)	<0.001
Oligoamnios (n/%)	Yes = 8 (6.90%) **	Yes= 1 (0.86%)	0.01
Polyhydramnios (n/%)	Yes = 0 (0%)	Yes= 5 (4.31%) **	0.02
PE related complications (n/%)	Eclampsia = 2 (1.72%) bruptio placentae = 3 (2.58%) HELLP syndrome = 3 (2.58%)	-	-
Newborn’s gender (n/%)	Male = 60 (51.72%) Female = 56 (48.28%)	Male = 59 (26.72%) Female = 57 (49.14%)	0.89
Gestational age at birth, weeks (mean ± SD)	35.87± 3.41 **	38.37 ± 1.40	<0.001
Mode of delivery (n/%)	Cesarean = 112 (96.55%) ** Vaginal = 4 (3.45%) **	Cesarean = 72 (62.07%) Vaginal = 44 (37.93%)	<0.001
Presentation (n/%)	Cephalic = 105 (90.52%) Breech = 10 (8.62%) Transverse = 1(0.86%)	Cephalic = 108 (93.10%) Breech = 8 (6.90%) Transverse = 0 (0%)	0.53
Apgar score at 1 min (mean ± SD)	7.25 ± 1.86 **	8.37 ± 0.88	<0.001
Apgar score at 5 min (mean ± SD)	7.98 ± 1.36 **	8.84 ± 0.71	<0.001
Birthweight, g (mean ± SD)	2519.91 ± 946.60 **	3240 ± 457.60	<0.001
Newborn’s length, cm (mean ± SD)	46.39 ± 6.00 **	50.79 ± 2.27	<0.001

Table 2 legend: PE—preeclampsia; SD—standard deviation; g—grams. Tests used: chi-squared for categorical variables, and *t*-tests for continuous variables; ** the data from which statistical significance originates.

**Table 3:** Comparison of paraclinical characteristics for the patients included in the analyzed subgroups.

Paraclinical Parameter	Subgroup 1 (EO-PE, n = 22)	Subgroup 2 (LO-PE, n = 94)	Sum of Squares	*p* Value	Subgroup 3 (Moderate PE, n = 88)	Subgroup 4 (Severe PE, n = 28)	Sum of Squares (SS)	*p* Value
MAP, MoM (mean ± SD)	1.13 ± 0.08	1.09 ± 0.11	0.02	0.15	1.11 ± 0.10	1.07 ± 0.11	0.02	0.13
UtA-PI, MoM (mean ± SD)	1.52 ± 0.27	1.54 ± 0.29	0.01	0.61	1.56 ± 0.27	1.47 ± 0.34	0.17	0.16
PAPP-A, MoM (mean ± SD)	0.82 ± 0.24	0.83 ± 0.58	0.001	0.94	0.77 ± 0.21	0.81 ± 0.21	0.02	0.43
PLGF, MoM (mean ± SD)	1.18 ± 0.51 **	0.85 ± 0.32	1.97	<0.001	0.86 ± 0.33	1.07 ± 0.47 **	1.04	<0.001
PP-13, MoM (mean ± SD)	0.94± 0.48 **	0.72 ± 0.23	0.80	0.003	0.74 ± 0.30	0.83 ± 0.33	0.14	0.21

Table 3 legend: PE—preeclampsia; SD—standard deviation; MoM—multiples of median; MAP—mean arterial pressure; UtA-PI—uterine artery pulsatility index; PLGF—placental growth factor; PP-13—placental protein-13; PAPP-A—pregnancy-associated plasma protein-A; EO-PE—early-onset preeclampsia; LO-PE—late-onset preeclampsia. Tests used: ANOVA analysis with the Bonferroni post hoc test; ** the data from which statistical significance originates.

**Table 4:** The predictive performance of machine learning-based models for the PE and its subtypes.

ML Model	Type of PE	TPR (%)	FNR (%)	PPV (%)	FDR (%)	Accuracy (%)	AUC Value	Precision	Recall	F1 Score
DT	All PE	94.1	5.9	91.4	8.6	92.8	0.93	0.91	0.94	0.93
	EO-PE	92.9	7.1	75	25	94.1	0.95	0.93	0.75	0.86
	LO-PE	66.7	33.3	92.9	7.1	88.2	0.80	0.93	0.93	0.93
	Moderate PE	75	25	91.7	8.3	82.4	0.80	0.85	0.92	0.88
	Severe PE	82.1	17.9	44.4	55.6	79.4	0.70	0.67	0.44	0.53
NB	All PE	96.3	3.7	96.4	3.6	98.6	0.98	0.96	0.96	0.98
	EO-PE	96.4	3.6	80	20	91.2	0.88	0.67	0.80	0.73
	LO-PE	33.3	66.7	87.1	12.9	85.3	0.72	0.96	0.87	0.92
	Moderate PE	25	75	79.3	20.7	73.5	0.68	0.88	0.79	0.84
	Severe PE	89.3	10.7	50	50	82.4	0.67	0.50	0.50	0.50
SVM	All PE	70.6	29.4	77.8	22.2	85.5	0.98	0.71	0.78	0.88
	EO-PE	96.4	3.6	80	20	91.2	0.91	0.67	0.80	0.73
	LO-PE	33.3	66.7	86.7	13.3	82.4	0.76	0.93	0.87	0.90
	Moderate PE	37.5	62.5	80.8	19.2	70.6	0.49	0.81	0.81	0.81
	Severe PE	85.7	14.3	20	80	73.5	0.64	0.17	0.20	0.18
RF	All PE	94.1	5.9	91.4	8.6	92.8	0.94	0.91	0.94	0.93
	EO-PE	92.9	7.1	71.4	28.6	91.2	0.94	0.83	0.71	0.77
	LO-PE	66.7	33.3	92.9	7.1	88.2	0.84	0.93	0.93	0.93
	Moderate PE	87.5	12.5	94.4	5.6	70.6	0.79	0.65	0.94	0.77
	Severe PE	85.7	14.3	33.3	66.7	76.5	0.76	0.33	0.33	0.33

Table 4 legend: All PE—all types of preeclampsia; EO-PE—early- onset preeclampsia; LO-PE—late-onset preeclampsia; ML—machine learning; DT—decision trees; NB—naïve Bayes; SVM—support vector machine; RF—random forest; TPR—true positive rate; FNR—false negative rate; PPV—positive predictive value; FDR—false detection rate; AUC—area under the curve.

**Table 5:** Analysis of variance among preeclampsia subgroups considering the predictive parameters from machine learning-based models.

Variance between Groups	Sum of Squares	Mean SQUARE	F	*p* Value	Eta Squared	95% CI Lower Bound	95% CI Upper Bound
TPR	4895.68	1631.89	12.25	<0.001	0.75	0.29	0.83
PPV	7455.69	2485.23	36.96	<0.001	0.90	0.67	0.93
Accuracy	546.05	182.01	13.68	<0.001	0.77	0.34	0.84
AUC	0.18	0.06	34.77	<0.001	0.89	0.65	0.93
Precision	0.64	0.21	11.41	<0.001	0.74	0.27	0.82
Recall	0.76	0.25	37.17	<0.001	0.90	0.67	0.93
F1 score	0.78	0.26	32.44	<0.001	0.89	0.63	0.92

Table 5 legend: TPR—true positive rate; PPV—positive predictive value; AUC—area under the curve; CI—confidence interval. Tests used: ANOVA analysis with the Bonferroni post hoc test.

## Data Availability

The data presented in this study are available on request from the corresponding author. The data are not publicly available due to local policies.
